# Proceedings of Societies

**Published:** 1871-07

**Authors:** Herbert Judd

**Affiliations:** Sec.; Galesburg, Ill.


### Proceedings of Societies.

GALESBURG, Ill., June 15, 1871.

The Sixth Annual Meeting of the Military Tract Medical Association was held in Masonic Hall, at Galesburg, Tuesday, June 13th.

M. Reece, of Abingdon, President, occupied the chair. Herbert Judd, of Galesburg, Secretary. Forty members were present, representing eight counties. During the morning service the following new officers were elected:

President—S. P. Breed, of Princeton. Vice President—John M. Morse, of Galesburg. Secretary and Treasurer—Herbert Judd, of Galesburg. Board of Censors—J. K. Crawford, of Monmouth; M. Reece, of Abingdon; Wm. Hamilton, of Galesburg

The address delivered by M. Reece, the retiring President, was voted to be spread upon the Record. The principal business was reports from the following standing committees:

*Essayists*—E. L. Phillips, of Galesburg; M. A. McClelland, of Knoxville.

*Surgery.* Wm. Hamilton, of Galesburg; J. N. Todd, of Kewanee, Geo. W. Crossley, Princeton.

*Practice of Medicine*—Hiram Vance, of Kewanee; W. L. Cuthbert, Monmouth; John P. McClanahan, Norwood.

*Materia Medica and Therapeutics*—J. P. Breed, of Princeton; H. Marshall, Monmouth; J. V. McCutchen, Norwood.

*Obstetrics and Diseases of Women and Children*—S. K. Crawford, of Monmouth; W. D. Sterling, Ionia; John W. Hensley, Yates City.

*Ophthalmology*—L. S Lambert, of Galesburg.

The cases of surgery, reported by Dr. Hamilton, were interesting. One case, an amputation of the arm near the shoulder, in which one of the ligatures (silk) still remained, the operation performed fifteen weeks since. In the discussion that followed, Dr. S. M. Hamilton, of Monmouth, recommended, from his practice, the silver wire ligature, cut off short, and left in the stump. He had always had good results, and healing by the first intention.

Upon the practice of medicine considerable matter of a general character was presented.

Dr. Wm. Byrns, of New Windsor, reported a man who took 200 grs. of powd. opium; he was some distance away, and when found was completely comatose. This case was successfully treated by emetics, strong coffee, cold effusions, and active moving of the patient.

President Breed read an extensive and valuable report upon Therapeutics. The reports from the Committee on Obstetrics were of a difficult variety, with one, a fatal result. Dr. L. S. Lambert read a lengthy paper upon "Anomalies of Refraction." This paper, together with the various reports were referred to the Committee on Publication.

Under miscellaneous business, considerable interesting matter was discussed.

Drs. Vance, S. M. Hamilton, Webster, Cuthbert and Gilbert presented some new rules in regard to membership and dues, which were adopted by the Association.

Dr. A. V. F. Gilbert, of Monmouth, was appointed by the President, as Committee on Necrology.

The deaths of Dr. H. C. Graham, of New Windsor, and Dr. Geo. Gillis, of North Prairie, were reported, and referred to this committee.

The meeting was thoroughly business-like and profitable to all.

Galesburg was chosen as the place for the next meeting.

Upon the motion of Dr. Hiram Vance, the Association voted that the Secretary forward a report of this meeting to the *Chicago Medical Examiner* for publication.

Dr. H. S. Hurd, of Galesburg, moved, which was voted, that the report be also forwarded to the *Chicago Medical Journal* for publication.

The Association then adjourned to meet at Galesburg, on the second Monday in January, 1872.

HERBERT JUDD, *Sec.*

